# An Internal Focus Leads to Longer Quiet Eye Durations in Novice Dart Players

**DOI:** 10.3389/fpsyg.2016.00633

**Published:** 2016-05-02

**Authors:** Sydney Querfurth, Linda Schücker, Marc H. E. de Lussanet, Karen Zentgraf

**Affiliations:** Institut für Sportwissenschaft, University of MünsterMünster, Germany

**Keywords:** internal and external attentional focus, gaze behavior, quiet eye, darts, inhibition hypothesis

## Abstract

While the benefits of both an external focus of attention (FOA) and of a longer quiet eye (QE) duration have been well researched in a wide range of sporting activities, little is known about the interaction of these two phenomena and how a potential interaction might influence performance. It was this study’s aim to investigate the interaction and potential effect on performance by using typical FOA instructions in a dart throwing task and examining both the QE and performance outcome. The results replicate neither the benefit of an external FOA nor the benefit of a longer QE duration. However, an interaction was observed, as QE was prolonged by an earlier onset and later offset in the internal focus condition only. As the typical effect of a performance benefit due to an external focus could not be replicated, the interaction must be interpreted with caution. The results are discussed and interpreted in light of the inhibition hypothesis and possible avenues for future research are suggested.

## Introduction

Attention is a complex, voluntary and selective process that has a limited capacity (Vickers, 2007; Gazzaniga et al., 2009). We can distinguish two different types of attention: The overt attention, which can be measured by head- or eye movements, or the covert attention, which is a shift in attention that cannot be observed and is purely mental (Posner, 1980). It is possible for these two systems to be controlled and shifted separately from each other (Posner, 1980; Posner and Raichle, 1994). That means the overt attention, the eye movements, may be directed toward one specific stimulus, while the covert attention is actually focused on something very different.

In general, visual attention and attentional abilities are very important for sports performance (Williams et al., 1999; Memmert et al., 2009). Athletes must be able to quickly perceive and assess (visual) information from their environment and process the relevant (visual) cues in order to swiftly react by generating the appropriate motor command and responding to the changing situation (Vickers, 2007). Some research has found little or no differences between athletes in general attentional or visual abilities (Memmert et al., 2009), while other research has found that top athletes and novice athletes show significant differences in their general attentional capacity and abilities: More specifically, the visual attention orientation ability of athletes was better than that of non-athletes (Kasper et al., 2012). It is important to fully understand visual abilities and attention in order to help athletes achieve their full potential.

When researching the role of attentional focus in sports performance, some have proposed to distinguish between an external and an internal focus of attention (FOA): “An internal focus is one that is directed at the performer’s own body movements, whereas an external focus is directed at the effects that his or her movements have on the environment” (Wulf, 2007, p. 4). The distinction between internal and external FOA generally addresses the covert attention. Current research on the FOA has found benefits for focusing one’s attention externally on the effect of the movement in the environment (Wulf, 2013). This benefit has been shown in a wide range of tasks: balance, precision, simple force production tasks (for an overview, see Wulf, 2013) as well as for endurance tasks (Schücker et al., 2013). In general, this advantage has been shown for experts and novices alike (Wulf, 2007; Schorer et al., 2012; see however, Beilock et al., 2002) and appears to be a benefit both for learning and for immediate performance (Wulf, 2013).

When researching the FOA it is important to use precise instructions, making it clear whether the overt or covert attention is being addressed. However, it must be noted that not all research has made a clear distinction between overt and covert attention, making it more difficult to compare and interpret results: In one study, examining a dart throwing task for instance, McKay and Wulf (2012) either instructed participants to focus on the Bull’s Eye, or the flight path of the dart. In doing this, they were trying to make a distinction between proximal and distal external focus in order to study the effect of a farther away external focus, but did not specify whether mental or visual attention was being addressed. Lohse et al. (2010) on the other hand instructed participants to visually focus on the Bull’s Eye and mentally focus on the flight of the dart, therefore making a distinction between visual (i.e., overt) and mental (i.e., covert) attention. This illustrates that it is very important to formulate the instructions in an unambiguous way, making clear whether overt or covert attention is being manipulated, in order to correctly interpret the results.

Apart from the benefits on the movement outcome, some studies have also examined qualitative aspects of the movements. Studies using electromyography (EMG) revealed enhanced neuro-muscular efficiency, showing that the movement economy can be improved by an external focus during a dart-throwing task (Lohse et al., 2010). In a simple force production experiment, Lohse and Sherwood (2012) found increased co-contraction in agonist and antagonist muscles for an internal FOA, suggesting internal focus on the muscles disrupts efficient patterns of muscle activation. Furthermore, it has been shown that an external FOA leads to more variability in relevant joint kinematics similar to functional variability in experts (Müller and Loosch, 1999; Lohse et al., 2010).

Specific effects of the FOA have allowed insight into the underlying mechanisms and help generate explanations as to why the external FOA is beneficial to performance. Most commonly, the constraint-action hypothesis is used to explain these findings (Wulf, 2007). According to this theory, an internal FOA constrains aspects of movement control that would otherwise be automatically executed. The explicit control of these movements leads to worse outcomes. An external focus of attention allows motor control to run automatically, leading to the best possible outcome (Wulf, 2007). This hypothesis might account for why the external focus leads to better outcomes, the reduced EMG activity and efficient neuromuscular activation under an external focus. The main points of criticism toward the constraint-action hypothesis are that it does not specify what is constrained within the motor system and that it cannot be embedded within other, larger theories of motor learning, for instance the control-based learning theory (Raab, 2007).

In another area of research, the so-called quiet eye (QE), has emerged as an important, achievement-defining factor in sports performance: The QE is the “final fixation or tracking gaze that is located on a specific location or object in the visuo-motor workspace within 3° of visual angle for a minimum of 100 ms” (Vickers, 2007, p. 11). On an intra-individual level, the QE of one person is longer on successful trials as compared to less successful trials (Vickers, 1996). On an inter-individual level, experts show significantly longer QE durations than novices (Vickers, 1996). The QE might be an indicator for the overt attention.

However, not only the length of the QE seems to be important, but also the duration in relation to functional aspects of the movement (Vickers et al., 2000; Klostermann et al., 2013). These data suggest that the optimal onset and offset are more functionally relevant than a long QE *per se* (Vickers et al., 2000). Klostermann et al. (2013) for instance showed that the length of QE had more impact if prolonged due to an earlier onset of the QE as compared to a later offset.

While the effect of the QE appears to be well established, the underlying mechanisms are less examined (for an overview of different theories, see Gonzales et al., 2015). In recent research, a new concept has emerged, the inhibition hypothesis (Klostermann, 2014; Klostermann et al., 2014). It suggests the QE period is used to find the optimal movement and that all other movement options must be inhibited prior to the movement execution. In general, when we perform a movement, there are many possible movement variants we could choose from. Our motor system plans the optimal movement variant and inhibits all other movement options. In order to plan the optimum movement, visual information must first be gathered. This happens during the QE period. As experts have a greater pool of movements to choose from, their QE period is longer than that of novices, who have a smaller movement repertoire. In this way, the Inhibition Hypothesis can explain why experts show longer QE durations than novices do (Klostermann et al., 2014). Visual information is gained during the QE period that is necessary to program the most efficient movement whereupon it is planned and all other alternative movement plans must be inhibited (for a similar neuroscientific concept, see the affordance competition hypothesis, Cisek and Kalaska, 2010).

While both the FOA as well as the QE have been much researched separately, they are rarely examined together. Both research areas follow different methodological approaches. When examining the FOA, most studies try to control eye movements by keeping them fixated to a specific location while manipulating attention through instruction or feedback (Wulf, 2007; Lohse and Sherwood, 2012). Researchers usually include instructions on gaze behavior (Lohse and Sherwood, 2012) but do not generally measure it. Research on the QE on the other hand examines the overt attention in various settings, without examining the covert attention (Vickers, 2007). Studies measure the gaze behavior, but do not assess what mental focus participants adopt. Therefore it was this study’s goal to examine the effect of typical focus of attention instructions, including instructions on gaze behavior (Lohse and Sherwood, 2012), on the QE. The aim is to examine the interaction of these two attentional phenomena, a goal that has only recently been pursued and demanded by researchers (Gonzales et al., 2015).

Some research has made assumptions about the interaction of overt and covert attention. For instance, Sherwood et al. (2014) examined the effect of FOA with blindfolded participants to fully exclude a potentially confounding effect of vision. As they still found a benefit of an external FOA, we can assume that directing one’s covert attention externally is, at least to some point, independent of a confounding effect of visual attention. However, some theories of visual perception and attention suggest visually focusing on the target automatically directs some attention externally to the target, thereby providing information necessary for motor programming (Henderson, 1996; Sherwood et al., 2014). This would suggest that during the QE period, some attention is directed externally toward the goal of the movement.

Furthermore some theories suggest that the prolonged QE duration is due to an external focus of attention (Vickers and Williams, 2007), or at least that the QE helps to improve performance by focusing attention externally (Moore et al., 2012). This would mean that during the QE period, attention is focused externally on the goal or effect of the movement and relevant information is gathered for optimal movement planning. As the focus is external, joint kinematics are not constrained and optimal outcome can be achieved. Other theories, however, suggest that longer flexion times in a dart-throwing task during an external focus are due to longer QE duration (Schorer et al., 2012). This would mean that while the attention is focused externally, the overt attention, i.e., the fixation, is fixed more stably on relevant cues and the information for optimal movement planning can be translated into motor execution without constraining joint dynamics via co-contractions for example.

Neither of these theories has been explicitly tested. In a recent study, addressing exactly this gap in the research, Klostermann et al. (2014) examined the QE during an internal and external focus of attention in a golf putting exercise. They examined both expert and near-expert golfers in their study, replicating typical FOA findings, i.e., both experts and near experts performed better under an external focus, as well as typical QE findings, i.e., experts showing longer QE durations than the near experts. While they found no differences in overall QE duration as a function of attention, they did find an interaction on QE offset: the difference in QE offset between experts and near experts was larger under the internal condition as compared to the external condition. Concerning the QE efficiency, a benefit of a longer QE period for performance was only found for experts and only under the internal focus condition: A prolonged QE duration was only beneficial to performance under the internal focus condition, not under the external condition. They interpret this finding in light of the inhibition hypothesis: During an external focus, attention is allocated toward goal dimensions and the movement is programmed automatically and without movement constraints. Therefore, there is little inhibition demand during the QE period. During an internal focus on the other hand, attention is allocated toward action specification in terms of movement variants, inducing movement programming under specific constraints in the motor system. Therefore, there is a greater inhibition demand, with a need for a prolonged QE period during which the movement variants are inhibited. This, in the end, might then induce benefits in performance.

Similar research, using experts, advanced players as well as novices, has been conducted by Rienhoff et al. (2014) on the relation between QE and focus of attention in a Basketball free throw. Although they were not able to replicate the benefit of an external focus of attention, they did find varying QE lengths in the internal and external focus conditions: Contrary to their hypothesis, Rienhoff et al. (2014) found shorter QE durations in the external focus condition as compared to the internal one. Their results showed that participants also performed poorer under an external focus on the ball. For all skill levels their results showed that a longer QE duration was associated with better shooting performance, regardless of focus instruction.

In Rienhoff et al.’s (2014) study, a longer QE was always associated with better performance, regardless of focus condition, while in Klostermann et al. (2014) study the functionality of QE varied between focus conditions (i.e., longer QE was only beneficial to performance under an internal focus). Regarding the overall QE duration, Rienhoff et al. (2014) found the QE duration was prolonged by an internal focus, while Klostermann et al. (2014) only revealed an interaction effect of expertise and focus on QE offset. It is necessary to gather further data on these phenomena to further examine and explain the relationship between focus of attention and QE. Therefore, the main aim of this study was to examine interactions of specific attentional focuses and the overt attention, the QE, while using typical focus of attention instructions used in other research. In their study, Klostermann et al. (2014) specifically altered the focus instructions to exclude instructions regarding gaze behavior. However, the focus of attention may be influenced by gaze instructions (Gonzales et al., 2015). Therefore it is the aim of this study to examine the effect of typical focus instructions including gaze instructions, in order to see what effect the covert attention has on eye movements, while controlling all other factors.

In this experiment a dart-throwing task was chosen, because both FOA studies and QE studies have used this task and therefore it should be easy to replicate results and compare them to existing research. Furthermore, both the effect of an external FOA (Lohse et al., 2014b) and the effects of longer QE duration (Vine and Wilson, 2011) have been shown with players with little experience, which is why this kind of participants was chosen for this study. In order to examine the interaction, it will first be necessary to reproduce the benefits of an external FOA as well as the benefits of longer QE durations.

## Materials and Methods

### Participants

Approval of the local university ethics committee was gained prior to the beginning of data collection. In order calculate optimal sample size, a power analysis was done using G^∗^Power 3.1. Based on an estimated large effect (*f* = 0.4) with the settings for the α-level to 0.05 and the power to 0.95 an optimal sample size of 18 participants was calculated. Data was collected from *N* = 22 subjects. Two subjects dropped out of the experiment during data collection because of technical difficulties while testing, leaving *N* = 20 (11 male and 9 female) subjects in the final participant group. The mean age of participants was *M* = 25.8 years (*SD* = 3.9) and all had normal or corrected-to-normal vision according to self-report. As measured by the Edinburgh Handedness Inventory (Oldfield, 1971), 18 participants were right handed (LQ-Score *m* = 88,23, *sd* = 21,24) and two were left-handed (LQ-Score *m* = -100, *sd* = 0). All subjects had some prior experience in dart throwing: 13 participants reported having played darts up to 10 times before, while the remaining seven participants reported having played more than 10 times. Participants were recruited through bulletins and social media postings and either received course credit or 8aaa for their participation.

### Apparatus and Measurements

The image of a standard sized dartboard was printed on paper, which was attached to a cork wall. The dartboard image was overlaid with a millimeter scale pattern for error collection. The Bull’s Eye was at a height of 1.73 m and throwing distance was 2.37 m in accordance with official tournament rules. Participants used regulation steel tip darts weighing 22 g. For every second participant a new printed dartboard was used. Performance was determined after participants completed a set of three throws. This procedure is in accordance with the standard competition conditions.

The movements of the head and fingertips were tracked using a passive reflective marker system (Qualisys, Gothenburg, Sweden). In order to track and capture the throwing motion the Qualisys Track Manager motion capture system was used (QTM, version 2.10). Ten OQUS 400 cameras were used, measuring at 400 Hz. Participants’ gaze behavior was measured using a wireless, head mounted system (Dikablis Mobile Eye Tracker 2.0, Ergoneers, Manching, Germany), measuring the pupil of the left eye at 25 Hz. The gaze direction was calibrated to the field camera’s image (equipped with a standard 4.3-mm objective) using a four-point method (d-lab 2.0). The computer running the Dikablis recording software (d-lab 2.0) sent the recorded gaze direction continuously via a local network to the computer running QTM. The position, orientation and delay of the gaze vector in the room was calibrated using the standard procedure of QTM (i.e., by fixating a passive reflective marker while wearing the eye tracker equipped with passive markers and moving the head in space).

### Procedure

Upon arrival in the laboratory, participants gave written informed consent and filled out the Edinburgh Handedness Inventory and a basic questionnaire on demographic information, dart experience, vision and general sport participation. After this, they proceeded with six sets of three throws as warm-up and baseline (18 darts total). For all throws, participants were instructed to aim for the bull’s eye and try to hit it as consistently as possible. Furthermore, they were instructed to visually focus on the bull’s eye. Next, the mobile eye-tracker was fitted onto participants, the standard calibration process of Dikablis was followed and the gaze vector was calibrated. In a last preparatory step, two reflective markers were attached to the thumb and forefinger of the throwing hand using double-sided sticky tape and additionally wrapping tape around the fingers. Participants then proceeded with another six sets of three throws (again, 18 total) as a second baseline so they could get used to wearing the eye-tracker.

The two experimental conditions, internal focus of attention and external focus of attention, consisted of two blocks of three sets of three throws each (18 throws in each condition total, broken up into two blocks of nine throws). Participants performed four blocks of alternating condition. The order of the blocks was counterbalanced between participants so that half of them started with the internal condition and the other half started with the external condition. Instructions for both conditions were taken from research by Lohse et al. (2010, p. 548) and translated into German by a native speaker. Again, participants were always instructed to “visually focus on the Bull’s Eye” (in German: “Fixiere mit den Augen das Bull’s Eye”) while mentally either focusing on the dart (external condition; in German: “während du dich auf den Flug des Dartes konzentrierst”) or their throwing arm (internal condition; in German “während du dich auf die Bewegung deines Armes konzentierst”). The instructions were repeated after each set of three throws. After the second baseline condition a gaze-check was conducted. If necessary the calibration-procedure was repeated. In total, the experiment took about 1 h for one participant.

### Data Analysis

#### Performance Data

The performance data was prepared and analyzed using Microsoft Excel 2007 and IBM SPSS 22. The dart throwing performance was measured for all 20 participants. The Radial Error (RE) was defined as the deviation from the Bull’s Eye in *X* and *Y* direction (cm).

(1) $${RE}_{i}=\sqrt{X_{i}^{2}+Y_{i}^{2}}\,$$

The Mean Radial Error (MRE) was calculated by averaging the RE of each subject’s throws for each condition (baseline 1, baseline 2, internal, and external focus) consisting of 18 (6 × 3) throws each. In addition to this, MRE was also calculated for each block of 18 throws, regardless of condition, in order to test an overall learning effect.

The Bivariate Variable Error (BVE) was calculated by using the distance of each throw from the average distance of all throws within a condition (*X*_c_ and *Y*_c_; Eq. 2). BVE was calculated for each condition (Hancock et al., 1995).

(2) $$BVE=\sqrt{\frac{1}{k}\overset{i}{\underset{i=1}{\Sigma }}{\left(X_{i}-X_{c}\right)}^{2}+{\left(Y_{i}-Y_{c}\right)}^{2}}\,$$

All data was tested (SPSS 22) for normal distribution using the Kolmogorov–Smirnov test, revealing a normal distribution for all data with all *p* > 0.05. Furthermore, Mauchly’s test for sphericity was calculated for each ANOVA. The results of Mauchly’s test will only be reported when significant and the degrees of freedom are corrected according to Greenhouse–Geisser.

For the MRE a one-factor univariate ANOVA with repeated measures was calculated with condition as the factor (four factor levels). Furthermore, a second one-factor univariate ANOVA with repeated measures was conducted in order to test an overall learning effect. For this, the throws were grouped into four temporally sequential blocks, regardless of the focus condition. Each block consisted of 18 throws: baseline 1, baseline 2, block 3 (18 throws of mixed focus), and block 4 (18 throws of mixed focus). As for the BVE, another one-factor univariate ANOVA was calculated with condition as the factor (four factor levels).

#### Eye Movement Data

Initial data preparation was done with QTM. The 3-D finger positions and the gaze vector data were exported in Matlab format. Further analysis was made using a self-made Matlab script (Matlab 2013b), while SPSS 22 was used for statistical analysis. Due to technical problems during data collection of three participants, the eye-tracking data could only be used for 17 of the 20 participants (in three subjects, the pupil was not detected reliably). From these 17 participants, another total of 15% of trials had to be excluded from further data analysis due to failed pupil detection. The excluded trials were distributed evenly across all conditions as revealed by a one-factor univariate ANOVA with condition as the factor [three factor levels; *F*(2,32) = 0.80, *p* = 0.46], so a cause for the high number of missing data systematically correlating with the experimental manipulation was not assumed.

The three-dimensional gaze vector contained *x* coordinates (describing the left–right axis), *y* coordinates (describing the front-to-back axis), and *z* coordinates (describing the ceiling-to-floor axis). This data was translated into two-dimensional coordinates on the plane of the dartboard, calculating the point of gaze on the board. In a next step, using the coordinates on the dartboard, the eye movements from one frame to the next were calculated using the Pythagorean Theorem, drawing a gaze path.

On the basis of the gaze path, the eye movements were segmented into saccades and fixations. Any eye movements exceeding 1.2° per frame (40 ms) were defined as saccade (1.2° corresponded to 4.9 cm on the dart board), while all smaller movements were considered to be part of a fixation. This criterion was based on the general distribution of eye movements (as measured by histograms of all eye movements of three randomly chosen participants) and is comparable to other QE research (Klostermann et al., 2014).

Quiet eye for a dart-throwing task can be defined as the “final fixation on the target with onset prior to the extension of the arm for the final throwing motion and offset when the fixation deviates off the target” (Vickers et al., 2000, p. 31). The movement initiation was defined as the first frame in which the markers on the fingers showed a positive velocity toward the dart board. The last saccade before this time was defined as QE onset, while the first saccade after the movement-initiation was defined as QE offset. The time elapsed in between was defined as the QE duration. The moment of the dart-release was determined as the first frame in which the distance between index finger and thumb increased.

By this method, QE duration, onset, and offset as well as moment of dart release were calculated for each throw and mean values were calculated overall, as well as for the baseline 2 and internal and external focus conditions. All QE data were measured in milliseconds. These data were tested for normal distribution using the Kolmogorov–Smirnov test, revealing a normal distribution for all data, with all *p* > 0.05. These data were entered into separate one factor univariate ANOVAs with repeated measures with condition as the factor (three factor levels). Again, Mauchly’s Test for sphericity was calculated for each ANOVA and will only be reported for significant tests and the degrees of freedom will in this case be corrected according to Greenhouse–Geisser.

To examine the QE efficiency correlations were calculated. In accordance with the methodology of Klostermann et al. (2014), correlations were calculated for the dart performance (as measured by the radial error) and QE data on each throw for all participants, for baseline 2, internal and external focus condition. This method reveals a functionality (or lack thereof) for each participant individually. Once a correlation for each person was calculated, the correlations were averaged and the resulting mean correlation was tested against zero using a one-sample *t*-test with zero as the test value. Pearson correlations were calculated between QE duration, onset, offset, and moment of dart release, respectively, with throwing performance (i.e., RE from the Bull’s Eye) on each throw for each participant. These correlations were then averaged for baseline, internal and external focus conditions, resulting in average correlations between dart performance and QE data.

## Results

**Table 1** presents the MRE, BVE, average QE duration, onset, offset, and time till dart release for each condition.

**Table 1:** Means (and standard deviations) for important dart performance variables and gaze data for both baseline and focus conditions.

	Dart Performance		QE Data			
	MRE^1^	BVE^1^	QE duration^2^	QE onset^2^	QE offset^2^	Time till dart release^2^
Baseline 1	8.68 (2.33)	8,83 (2.39)	/	/	/	/
Baseline 2	8.45 (1.64)	9.16 (1.98)	880 (350)	770 (340)	100 (40)	–80 (50)
Internal	8.53 (2.35)	8.67 (2.58)	1300 (480)	1170 (490)	130 (80)	–60 (70)
External	8.25 (1.89)	8.46 (2.05)	1040 (460)	940 (460)	100 (40)	60 (60)

*^1^Data based on N = 20, ^2^Data based on N = 17; MRE, Mean Radial Error (cm); BVE, Bivariate Variable Error (cm); QE, quiet eye (ms); Baseline 1 = first baseline, without gaze tracking data; Baseline 2 = second baseline with gaze tracking data; (A negative value in “Time till dart release” indicates that the dart was released after the end of the fixation while a positive value indicates the number of seconds before the end of the fixation the darts were released). Values given in brackets are standard deviations.*

**FIGURE 1:**
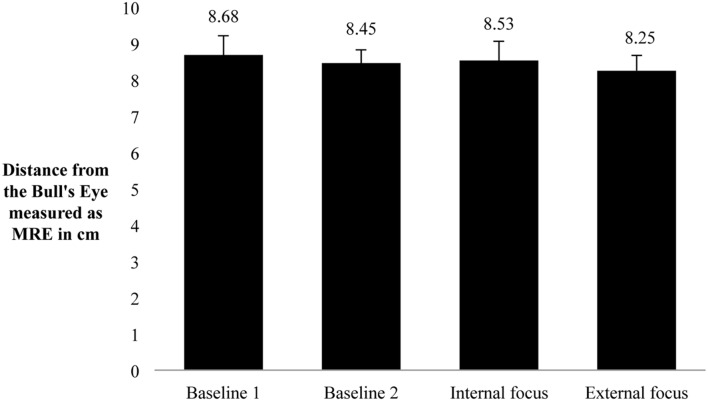
**Mean Radial Error (MRE) of dart performance for each condition.** For baseline 1 (without gaze tracking), baseline 2 (with gaze tracking), internal and external focus conditions; error-indicators depicted are standard errors of the mean.

As depicted in **Figure 1**, the ANOVA revealed no effect of focus of attention on dart throwing performance for the MRE [*F*(3,57) = 0.37, *p* = 0.78, η^2^ = 0.02]. Furthermore, the second ANOVA revealed no significant difference between the blocks [*F*(3,57) = 0.94, *p* = 0.43, η^2^ = 0.05] indicating that performance did not change in the course of the experiment as a function of experience. The ANOVA with BVE revealed no significant effect as a function of condition [*F*(3,57) = 0.84, *p* = 0.48, η^2^ = 0.04].

When examining the QE efficiency, mean correlations were examined. In the case of QE duration, for example, a high negative correlation would suggest that longer QE durations were associated with shorter distances from the bull’s eye (i.e., better performance), a high positive correlation that longer QE durations were associated with farther distances from the bull’s eye (i.e., worse performance) and no correlation suggests there is no connection between QE and throwing performance. As can be seen in **Table 2**, no significant correlations between QE duration, onset, offset, or time till dart release and throwing performance measured as RE were revealed. All correlations were between -0.04 < *r* < 0.08 with all *p* > 0.05.

**Table 2:** Mean correlations between dart performance variables and QE-Data.

	Onset and RE	Offset and RE	Duration and RE	Time till dart release and RE
Baseline 2	-0.04	0.05	0.01	0.05
Internal	0.03	0.05	0.02	0.01
External	-0.08	0.08	0.01	-0.10

*RE, radial error, measured in cm; QE, quiet eye, all QE data were measured in ms.*

**FIGURE 2:**
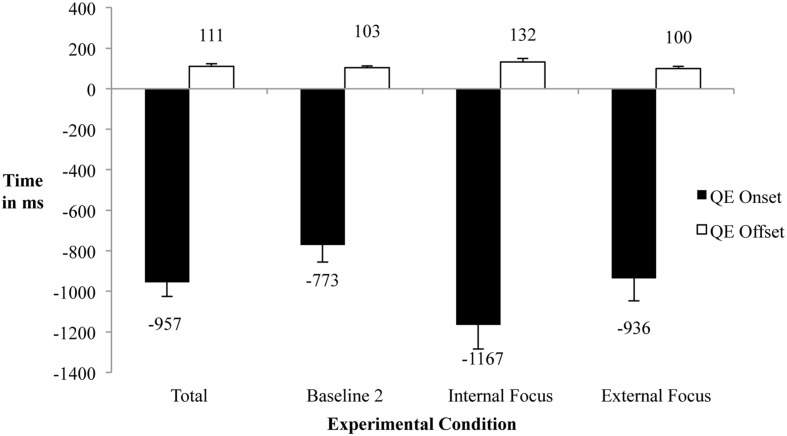
**QE onset, offset, and duration for each condition and overall.** Measurement in milli-seconds; 0 is the first moment the throwing arm accelerates toward the dart board; error-indicators depicted are standard errors of the mean.

As seen in **Figure 2**, a significant main effect of condition was revealed for QE duration [*F*(2,32) = 5.31, *p* < 0.05, η^2^ = 0.25], QE onset [*F*(2,32) = 4.44, *p* < 0.05, η^2^ = 0.22] and QE offset [significant Mauchly’s test *W*(2) = 0.59, *p* < 0.05, *F*(1.42,22.66) = 5.08, *p* < 0.05, η^2^ = 0.24]. Bonferroni corrected *post hoc* tests revealed that participants had the longest QE durations, earliest onset and latest offset in the internal focus condition as compared to the baseline, with all *p* < 0.05.

## Discussion

Although several studies have examined the interaction of FOA and QE, their contradictory findings do not yet provide satisfactory answers (Klostermann et al., 2014; Rienhoff et al., 2014). Therefore, it was this study’s aim to further investigate the interaction and potential effect on performance. Furthermore it was important to use typical focus of attention instructions, differentiating between visual and mental attention and including an instruction for gaze behavior, and examine both QE and performance outcome.

In order to be able to examine the interaction, as was the main focus of this paper, one must first replicate the typical findings of the focus of attention and QE research. Many studies find that an external focus of attention is beneficial when compared to an internal focus of attention (Wulf, 2013). In this study, however, we found no differences in dart throwing performance with respect to attentional focus. It has also been found that an external focus is related to greater variability in joint kinematics and less variability in the goal dimension (Lohse et al., 2010). However, our results showed no reduction in variability as a function of attentional focus. To further examine the dart throwing performance, the Variable Error was calculated for the *X* and *Y*-axis individually, in order to test the plausibility of the data. *T*-tests comparing the variance in *X* and *Y* directions revealed that the variability was greater for the *Y*-axis than for the *X*-axis in all conditions, although the difference was only significant for the baseline 2 [*t*(19) = -2.83, *p* < 0.05] and the internal focus condition [*t*(19) = -2.41, *p* < 0.05]. This pattern is as can be expected for this kind of task (Müller and Loosch, 1999; van Essen et al., 2010), confirming the plausibility of the data. Furthermore, the data was tested for an overall learning effect. The ANOVA examining the dart performance from the first set of 18 throws to the last set of 18 throws revealed that participants’ performance did not vary during the course of the experiment.

While a benefit of the external focus of attention has been replicated many times and is well established, some studies do not find this general beneficial effect (Beilock et al., 2002; Zentgraf and Munzert, 2009; Schorer et al., 2012). Wulf (2013) argues that most studies failing to replicate the benefit of an external focus do so due to methodological mistakes. However, these were taken into account for this study’s design and the instructions were replicated from other focus of attention research (Lohse et al., 2010).

The second aim of this study was to replicate typical research findings concerning the QE. These include that a prolonged QE duration typically coincides with better targeting performance, but also that the onset and offset relative to movement initiation are important. That is, for closed-loop performance, where movement feedback is given during the movement, especially a late QE offset is important as visual information can continually be perceived and processed (Klostermann et al., 2014). In open-loop tasks, where no proprioceptive movement feedback can be processed for changing movement parameters (as is the case in the dart throwing task), gathering visual information early on in the movement is of special importance (Vickers et al., 2000). However, this study was not able to replicate these findings: The results show no correlation between QE duration, onset, offset, or time till dart release with the MRE in the dart task.

The main interest of this paper was to investigate the interaction between the FOA and the QE, a goal that has only recently been pursued in research. So far, studies examining this interaction have found effects of the focus instruction on the QE duration, prolonging QE under an internal focus (Rienhoff et al., 2014) or that the focus instruction influences QE efficiency (Klostermann et al., 2014).

In the present study, QE was longest in the internal focus condition. In this condition, QE was prolonged both through an earlier onset and a later offset when compared to baseline condition. The difference between internal and external focus conditions did not reach significance. Furthermore, as shown above, no correlation between QE durations and throwing performance could be found. These results are similar to those found by Rienhoff et al. (2014) reporting prolonged QE durations under an internal focus of attention. However, in their study, Rienhoff et al. (2014) did find that a longer QE was generally associated with better performance as hits were always accompanied by longer QE durations than misses were, regardless of focus condition. Klostermann et al. (2014) on the other hand, found no difference in the total duration of the QE between their two focus conditions, but found that the difference in QE offset between experts and near experts was larger under the internal condition. Most interestingly, a longer QE only benefitted performance under an internal focus of attention. Under an external focus of attention, a longer QE was not related to better performance.

In their paper, Klostermann et al. (2014) argued that their findings support the inhibition hypothesis as the internal focus condition has a greater inhibition demand. The results of the present study might also be seen as corroborating the inhibition hypothesis. However, this is a *post hoc* finding since it was not our goal to test the inhibition hypothesis. Nonetheless, the QE period was prolonged during the internal focus condition. One might speculate that this could be due to a greater inhibition demand compared to the external focus condition. Future research may be designed to explicitly test predictions made by the inhibition hypothesis and to determine why in Klostermann et al.’s (2014) study the functionality of the QE was affected, i.e., a longer QE was only beneficial to performance under an internal focus, while in the present study, as well as in the study by Rienhoff et al. (2014) the duration of QE appears to be influenced by the FOA instruction.

The results of this study, however, must be interpreted with caution, as neither the benefit of an external focus, nor the functionality of the QE could be replicated. There are other research findings that question the functionality of the QE: de Oliveira et al. (2006) for instance experimentally manipulated the QE duration by occluding vision in a basketball jump shooting task and found no performance detriments due to short QE durations. Furthermore, Glöckner et al. (2012) argue that not only the last fixation, but rather the shift of attentional focus over a longer time is important to predict the outcome in handball plays. However, it is questionable whether findings from such dynamic, more closed-loop activities as basketball jump shooting or handball plays are applicable to the open-loop task of dart throwing. However, Klostermann et al. (2014) also found a general functionality of QE in their experiment when examining a golf-putting exercise which is more similar to the dart throw.

Vickers et al. (2000) found that while the fixation does not necessarily need to be maintained after movement initiation, QE offset occurring too early in the movement leads to decreased performance. Furthermore, new research suggests that even for open loops tasks (like dart throwing or golf putting) a late QE offset is important, gathering information late in the process (Vine et al., 2014, 2015). In general, participants in the present experiment had very early QE offsets, often making a saccade at the moment of movement initiation. It is therefore possible that the QE simply ended too soon to reveal a functional relationship, between QE offset and MRE in the dart performance.

Another explanation might be in the participants’ level of experience. Rienhoff et al. (2014) found no difference in QE durations for hits or misses in a basketball free throw task for novice players. Similarly, some research suggests that the benefit of an external FOA is predominantly evident in experts and often cannot be replicated in novice performers, or even that novice players benefit from an internal focus (Beilock et al., 2002; Perkins-Ceccato et al., 2003; Wulf, 2013). However, the participants selected for the present study were fairly inexperienced but better than novices. Their overall performance was similar to that of the participants in other studies: For example, Lohse et al. (2010) found a benefit of an external focus of attention using participants who had an absolute error from the bull’s eye of *m* = 8.06 cm. Vickers (2007) examined QE in dart players who had an average of *m* = 5.2 cm distance from the bull’s eye. Furthermore, as revealed by an ANOVA, overall performance did not change in the course of the experiment. If the participants had been novices, one would expect a learning curve in the course of the experiment.

Some research suggests that the participants’ preference or expectations as to which condition is beneficial to performance might influence the effect of the attentional focus (Lohse and Sherwood, 2011). Further research has shown that especially novice performers tend to prefer an internal focus of attention (Wulf and Su, 2007; Marchant et al., 2009). Even though, great care was taken to not bias participants toward one focus condition many verbally reported preferring the internal focus condition to the external one. However, McKay and Wulf (2012) reveal that an external focus of attention is beneficial even if an internal focus is preferred. Even so, an effect of participants’ expectations cannot be ruled out.

A further issue might be that participants were distracted by the eye- and motion-tracking equipment they were wearing. Even though the eye-tracking glasses were not detrimental to overall performance (as can be seen when comparing the MRE in the first and second baseline), it may well have affected the participants’ attentional focus. When testing learning and transfer effects of attentional focus, Lohse et al. (2014a) had participants wear sleeves with weights in them on their throwing arms. This reduced the benefits of an external focus. Presumably, the changed dynamics of the limbs hindered the participants in complying with instructed attentional focus (Lohse et al., 2014a). Similarly the tape over the finger and thumb used to attach the reflective markers may well have affected the way participants were able to feel the dart. While this did not have a detrimental effect on their overall performance, it might well have interfered with their ability to adopt the attentional focus. Almost all participants reported being affected by the tape. This may have been a salient stimulus directing attention bottom-up to the source of irritation and therefore hindering or impairing compliance with instructed attentional focus. Overall, it is a limitation of this study that no manipulation check was conducted, which would have revealed participants’ difficulties adopting the attentional focus. However, it is a general problem of focus research that no good manipulation check exists (Schorer et al., 2012).

Furthermore, it is important to point out one limitation of this study, that can also be found in other QE research: The eye-tracking system. In this study, the Dikablis System was chosen, because it can be integrated into the motion capture system, thereby allowing the differentiation of eye- and head movements. It is a light, mobile eye-tracking system that allows for very natural movements and hardly hinders the wearer. However, with a measuring rate of 25 Hz, the temporal acuity is fairly low. This was not seen as a problem, because other studies examining the QE also used systems measuring with similar measuring rates (Vickers et al., 2000 measuring with 30 Hz; Vickers and Williams, 2007 measuring with 30 Hz; Klostermann et al., 2013 measuring with 25 Hz). However, this only allows the detection of eye-movements in intervals of 40 ms. Furthermore, when compared to the 400 Hz measuring rate of Qualisys, rounding errors are likely when calculating the QE onset and offset in relation to the moment of movement initiation. The use of an eye-tracking system with higher temporal acuity would therefore be desirable for future research.

Another limitation is that current research shows no uniformly used definition for QE, with some researchers defining QE within 3° of visual angle (Vickers, 2007), while others define it within only 1.2° of visual angle (Klostermann et al., 2014) The criterion used in this study therefore was a plausibility criterion, based on other studies examining QE (Klostermann et al., 2014) as well as on the general distribution of eye movements as detected by the Dikablis system. However, future research should focus on finding a uniformly used definition of QE, as the definition of QE can greatly impact the outcome of research. For instance, in a dart-throwing task with a typical throwing distance of 2.37 m, the difference in definition leads to eye movements on the dartboard from 5 cm to almost 15 cm both counting as QE. A typical Bull’s Eye has a diameter of only 1 cm. It is doubtful that looking at the dartboard within a circle of 15 cm should be helpful at hitting a target only 1 cm in diameter. Comparing QE as defined by differing fixation definition could be one avenue for future research, examining exactly how large the area of the QE must be in order to find a meaningful and functional relationship between QE and performance.

Besides this future research could further investigate the variability in joint kinematics. As discussed, an external focus of attention leads to more variability in joint kinematics, while reducing variability in the outcome dimension, thereby improving accuracy. This study found no differences in variability in goal dimensions. However, it would be interesting to take a look at the corresponding variability in shoulder and elbow angle, angular velocity and location of the hand at dart release. Investigating not only the relationship between FOA and QE with respect to the outcome, but also examining qualitative aspects of the movement such as neuromuscular efficiency or variability in joint kinematics (Zentgraf and Munzert, 2009) could further uncover the underlying mechanisms and possible connections between the two constructs.

This line of future research could further be advanced by expertise research: The increased variability in joint kinematics found under an external focus is similar to functional variability found in experts: Experts in a specific sport show low variability in outcome dimension, with very high accuracy, while they are fairly variable in joint kinematics of the movement (Müller and Loosch, 1999; Lohse et al., 2010). At the same time, research has compared the visual-perceptual and attentional abilities of elite and novice athletes. Some research has found little or no differences between athletes in general attentional or visual abilities (Memmert et al., 2009), while other research has found that top athletes and novice athletes show significant differences in their general attentional capacity and abilities: More specifically, the visual attention orientation ability of athletes was better than that of non-athletes (Kasper et al., 2012; for a meta-analytic review supporting specific attentional superiority in athletes, see Voss et al., 2009). In regards to elite athletes’ visual abilities, research shows they generally use fewer fixations of longer durations, including longer QE durations (Mann et al., 2007). By researching the relationship between visual perceptual abilities and variability in goal dimensions and joint kinematics in expert and novice players new insights into both the QE and attentional focus processes can be gained.

Furthermore, research should take a closer look at QE in different sports and investigate how comparable these phenomena really are. Open- and closed-loop tasks have very different attentional demands and therefore it may well be that the functionality of QE is quite different for these tasks. Drawing conclusions from one sport or task to another may very well be generalizing across very different processes.

## Conclusion

This study’s aim was to examine possible interactions between FOA and QE. The results offer evidence in line with the Inhibition Hypothesis as postulated by Klostermann (2014) in that the prolonged QE duration during the internal focus might be due to a greater number of movement variables to be inhibited. Failing to replicate basic FOA or QE results, however, the results of the interaction must be interpreted with caution. Overall this study shows that it is important to consider QE effects when examining focus of attention and also to consider effects of the focus of attention when examining the QE. It is important for future research to further investigate this relationship between FOA and QE to more fully understand both concepts. Furthermore, clear definitions are essential for finding consistent and comparable results.

## Author Contributions

SQ made substantial contributions to the conception of the work, the acquisition, analysis and interpretation of data, as well as drafting and revising the manuscript. LS made substantial contributions to the conception of the work, as well as the analysis and interpretation of data and in revising the manuscript. ML made substantial contributions to the acquisition, analysis and interpretation of data as well as revising the manuscript. KZ made substantial contributions to the conception of the work, as well as the analysis and interpretation of data and in revising the manuscript. All authors gave approval of the final version.

## Conflict of Interest Statement

The authors declare that the research was conducted in the absence of any commercial or financial relationships that could be construed as a potential conflict of interest.
